# Dendritic Fibromyxolipoma of the Pyriform Sinus: A Case Report and Review of the Literature

**DOI:** 10.1155/2016/7289017

**Published:** 2016-12-18

**Authors:** Abdulrahim AlAbdulsalam, Maha Arafah

**Affiliations:** ^1^Section of Pathology, Department of Biomedical Sciences, College of Medicine, King Faisal University, Al-Ahsa, Saudi Arabia; ^2^Section of Anatomic Pathology, Department of Pathology and Laboratory Medicine, College of Medicine, King Saud University, Riyadh, Saudi Arabia

## Abstract

Dendritic fibromyxolipoma is a rare and distinctive soft tissue neoplasm that is considered by many authors as a variant of spindle cell lipoma and characterized by the presence of dendritic cytoplasmic processes, plexiform vascular pattern, and keloidal collagen. It has never been reported in the larynx and hypopharynx. Its rarity and the potential to mistake it as a more clinically aggressive myxoid soft tissue neoplasm highlight the importance of its recognition. Here, a case of a dendritic fibromyxolipoma of the pyriform sinus in a 38-year-old male who presented with dysphagia, change of voice, and stridor is reported. A review of the literature, including histopathologic features and differential diagnosis, is also included.

## 1. Introduction

Lipomas are the most common soft tissue tumors in adults. Spindle cell lipoma (SCL) is an uncommon variant that has a characteristic clinical setting. It usually presents as a mass in the posterior neck and shoulder region of men between the ages of 40 and 50. It has also been described in many other locations. About 7 cases have been reported in the larynx and hypopharynx. Dendritic fibromyxolipoma (DFML) is a rare and distinctive tumor that is considered by many as a variant of spindle cell lipoma characterized by extensive myxoid change and the presence of stellate cells with dendritic processes. A total of 21 cases have been reported, none of which are in the larynx and hypopharynx. Here, we describe a case of dendritic fibromyxolipoma of the pyriform sinus, with review of the literature.

## 2. Case Presentation

A 38-year-old male who is known to be a heavy smoker for 10 years presented with progressive dysphagia for one year, associated with change of voice, stridor, and sleep disturbance in the last three months. He has no past history of trauma or surgery in the head and neck and no family history of cancer. Laryngoscopic examination showed a large oval mass originating from the left pyriform sinus and causing narrowing of both the hypopharynx and larynx. The clinical impression was that of a retention cyst.

**Figure 1:**
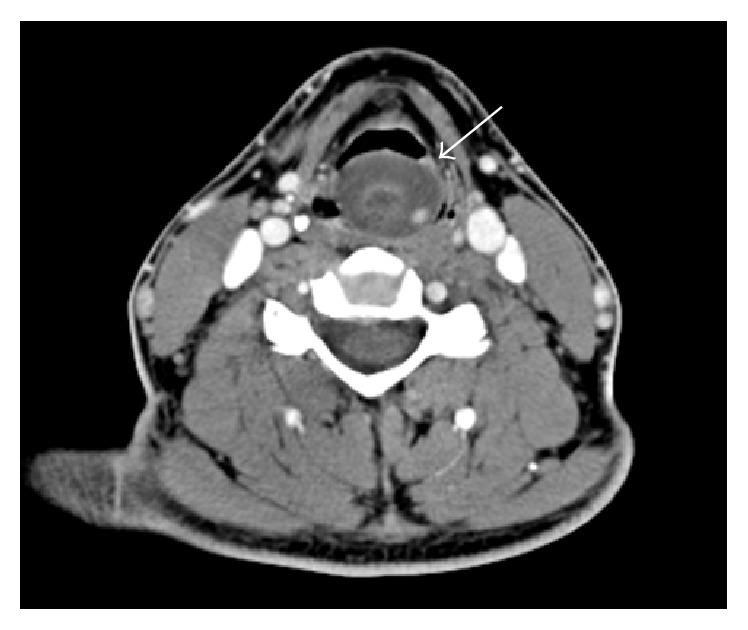
Contrast-enhanced CT scan of the neck. A well circumscribed, focally enhancing mass is noted in the left pyriform sinus (arrow).

Contrast-enhanced CT scan of the head and neck was performed. It confirmed the presence of the mass in the hypopharynx, measuring 3.4 × 3.4 × 2.8 cm. It was well-defined, hypodense, mostly homogenous, and nonenhancing, with a very focal area of contrast enhancement (Figure 1).

**Figure 2:**
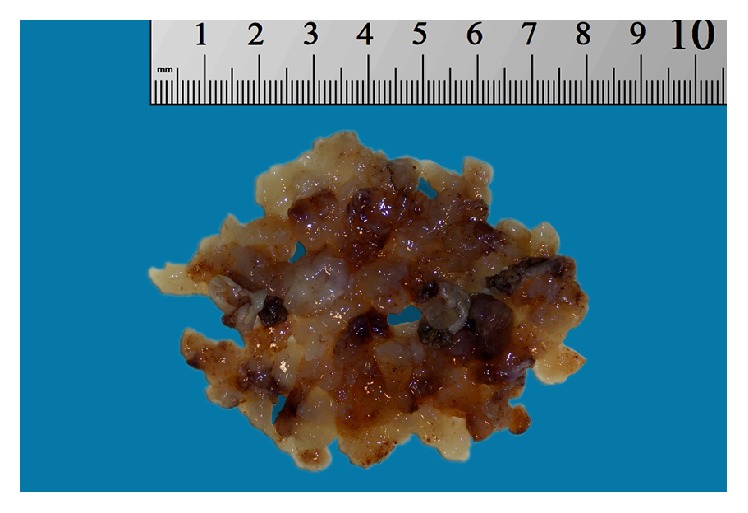
Gross appearance of the specimen.

A microlaryngoscopy with debulking of the mass by CO_2_ laser was performed. Gross examination revealed multiple pieces of soft yellow-tan and glistening tissue measuring 7.5 × 6.5 × 0.5 cm in aggregate (Figure 2).

**Figure 3:**
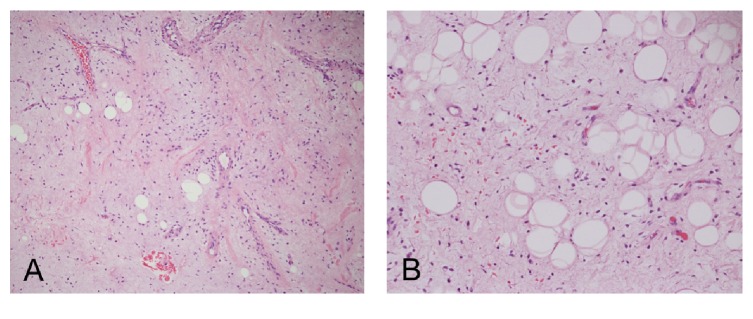
(A) Hematoxylin and eosin- (H&E-) stained sections showed a hypocellular neoplasm with myxoid background, ropy collagen bundles, and plexiform blood vessels (100x). (B) Mature adipocytes with scattered bland spindle cells (400x).

**Figure 4:**
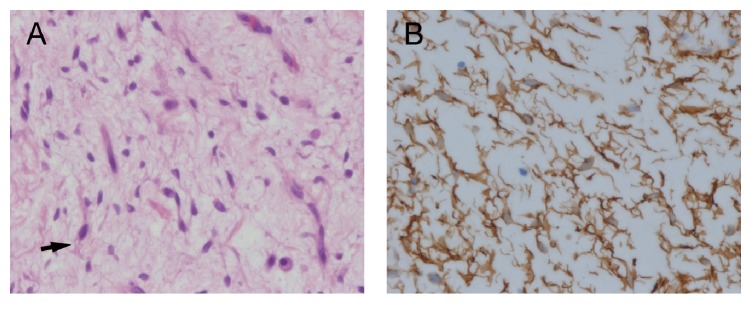
(A) H&E-stained section showed the dendritic cytoplasmic processes of the spindle cells (arrow) (600x). (B) Immunohistochemistry for CD34 also highlights the dendritic cytoplasmic processes.

On microscopic examination, the pieces are composed of a hypocellular spindle cell neoplasm with abundant myxoid background and plexiform blood vessels (Figure 3(A)). Few mature adipocytes are present as single cells or as small clusters scattered throughout the lesion (Figure 3(B)). The spindle cells have a stellate shape with small oval nuclei, inconspicuous nucleoli, and multiple dendritic cytoplasmic processes (Figure 4(A)). Some areas have a more fibrotic background with thick (ropy) collagen bundles. Numerous mast cells were also present. There were no lipoblasts and no appreciable mitotic activity in the lesion. Immunohistochemistry showed that the spindle cells were strongly positive for vimentin, CD34, and Bcl-2 and negative for desmin, alpha smooth-muscle actin, and S-100 protein. The dendritic cytoplasmic processes were highlighted by CD34 (Figure 4(B)) and vimentin. Ki-67 labeling index was very low. The adipocytes were positive for S-100 protein.

## 3. Discussion

DFML has been initially described by Suster et al. in 1998 in a 12-case series report [1]. Since then, about 9 additional cases have been reported in the English literature and cited in PUBMED (Table 1) [2–10]. The age ranges from 24 to 81 years, with a median age of 65 years. Male to female ratio is 4 : 1. The most common locations are the subcutaneous tissue and muscular fascia of the shoulder, neck, and back. Other reported locations include the nasal tip, lip, forearm, and intramuscular location. The lesion is usually well-circumscribed and slow growing, with a gelatinous gray to yellow cut surface.

Microscopically, it is composed of bland-appearing spindle cells with abundant myxoid background, prominent anastomosing blood vessels, areas of ropy collagen deposition, and prominent mast cells. The cells have oval nuclei and contain multiple dendritic cytoplasmic processes that are best highlighted by immunohistochemistry for CD34 and vimentin. CD99 positivity has been reported in a case [3]. S-100 is negative.

There are two opinions regarding the nature of this lesion. The first is that it represents a peculiar variant of spindle cell lipoma with extensive myxoid change. Evidence supporting this claim includes the similarity of clinical characteristics including age, gender, and location, similar morphologic features, and immunophenotype with identical clinical outcome. Wong et al. [6] demonstrated the presence of 13q14.3 deletion in one case of DFML, a recurrent finding in SCL. We speculate that many cases previously diagnosed as myxoid lipoma or myxolipoma belong to the category of DFML. DFML is morphologically distinguished from spindle cell lipoma by the presence of dendritic cytoplasmic processes, plexiform vascular pattern, and abundant keloidal collagen.

The other view is that it represents a tumor with combined features of SCL and solitary fibrous tumor (SFT) or an intermediate form between these two entities [3, 9]. Evidence supporting this view includes the similar immunohistochemical profile (positivity for CD34, Bcl-2, and CD99 in some cases). In addition, myxoid change has been reported in SFT [11]. However, SFT lacks the adipose tissue component of DFML and has prominent hemangiopericytoma-like vessels.

DFML can be confused with other more clinically aggressive neoplasms, including myxoid liposarcoma (MLS), low-grade fibromyxoid sarcoma (LFMS), and myxofibrosarcoma, especially the low-grade type, highlighting the importance of its recognition. MLS typically develops in the deep soft tissues of lower extremities. It shares many features with DFML including plexiform vascular pattern and abundant myxoid matrix. However, the lack of lipoblasts and atypical cells, negativity for S-100 and positivity for CD34, differentiates it from DFML. In addition, about 95% of cases of MLS harbor a recurrent translocation involving DDIT3 gene at chromosome 12 [12]. LFMS is composed of alternating fibrous and myxoid areas with low to moderate cellularity, bland spindle cells, and a characteristic swirling, whorled growth pattern [13]. It shows positivity for MUC-4 by immunohistochemistry in virtually all cases [14]. Myxofibrosarcoma is characterized by presentation in elderly patients and consists of a hypocellular neoplasm with myxoid background. It is distinguished by the presence of atypical and hyperchromatic cells and curvilinear blood vessels [15].

Follow-up data on all available reported cases showed no recurrence or metastases, with periods ranging from 4 months to 13 years.

In conclusion, DFML is a rare tumor that shares many similarities with spindle cell lipoma. This study presents the first case reported in the hypopharynx. It is important to be aware of this lesion in order to not misdiagnose it as other clinically more aggressive neoplasms such as myxoid liposarcoma and low-grade fibromyxoid sarcoma. More studies including molecular testing for 13q deletion and NAB2-STAT6 fusion [11] in cases of DFML would help to accurately identify the nature of this neoplasm.

## Figures and Tables

**Table 1:** Clinical findings of all reported cases of DFML.

Number	Author	Year reported	Age	Sex	Location	Size (cm)	Follow-up
1	Suster [1]	1998	33	M	Left posterior shoulder, acromion region	11	NA
2			54	M	Right posterior neck	5	NA
3			58	M	Right shoulder	7.5	7 years, NRM
4			63	M	Upper back	6	NA
5			66	M	Back of neck	8	NA
6			66	M	Posterior axillary fold	9	NA
7			70	M	Right nasal area	2	11 years, NRM
8			73	M	Right posterior neck	7	13 years, NRM
9			77	M	Back of neck	3	5 years, NRM
10			79	M	Right chest wall	3.5	Died of metastatic lung cancer
11			81	M	Left chest wall	3.5	5 years, NRM
12			50	F	Right upper back	6	NA
13	Karim [2]	2003	73	M	Right shoulder, between infraspinatus and deltoid muscles	13	8 months, NRM
14	Maskery [3]	2011	36	F	Lower lip	2	2 years, NRM
15	Dahlin [4]	2012	65	F	Left forearm, adherent to median nerve	3.2	NA
16	Zhang [5]	2013	32	F	Right inguinal and perineum region	24	9 months, NRM
17	Wong [6]	2014	67	M	Left shoulder	7	4 months, NRM
18	Han [7]	2014	69	M	Nasal tip	1	NA
19	Xu [8]	2015	24	M	Left shoulder, triceps brachii (intramuscular)	14	4 years, NRM
20	Liu [9]	2015	53	M	Right back, latissimus dorsi (intramuscular)	2	1 year, NRM
21	Cilogu [10]	2016	59	F	Left inguinal region	17	3 years, NRM

M, male; F, female; NA, information not available; NRM, no recurrence or metastasis.
